# Experimental study and clustering of operating staff of search systems in the sense of stress resistance

**DOI:** 10.3389/fdata.2023.1239017

**Published:** 2023-10-23

**Authors:** Nataliya Shakhovska, Roman Kaminskyi, Bohdan Khudoba

**Affiliations:** Department of Artificial Intelligence, Lviv Polytechnic National University, Lviv, Ukraine

**Keywords:** human-machine, operator personnel, decision-making, clustering algorithm, stress management

## Abstract

**Introduction:**

The main goal of this study is to develop a methodology for the organization of experimental selection of operator personnel based on the analysis of their behavior under the influence of micro-stresses.

**Methods:**

A human-machine interface model has been developed, which considers the change in the functional state of the human operator. The presented concept of the difficulty of detecting the object of attention contributed to developing a particular sequence of ordinary test images with stressor images included in it and presented models of the flow of presenting test images to the recipient.

**Results:**

With the help of descriptive statistics, the parameters of individual box-plot diagrams were determined, and the recipient group was clustered.

**Discussion:**

Overall, the proposed approach based on the example of the conducted grouping makes it possible to ensure the objectivity and efficiency of the professional selection of applicants for operator specialties.

## 1. Introduction

In modern information and search automated systems, the main role is played by the human-machine interface for decision-making based on the detection of objects of a given class in the information field. The human operator, as an element of such an interface, carefully reviews the provided information on the information field—the monitor screen, analyzing images of scenes, for example, pictures of territories, economic tables, abstract data, etc. Based on the obtained results of the analysis, the operator forms or chooses from a set of alternatives the appropriate decision, for which he mostly bears some responsibility. Such, let's call them primary solutions, can be both the final and the basis for further functions of this search engine. However, among the factors that in one way or another can negatively affect the quality of the work of this interface, one of the first places is stress in the form of a collection of micro stresses.

Due to the certain complexity of the provided images and their large number and variety, the functional state of a person becomes nervously tense, close to stressing, and in most cases this state is micro-stress. Individually, micro-stresses may be imperceptible to a person, but in the aggregate, their effect can be quite noticeable.

One such source of micro-stress in the field of information search can be a sudden change in the information flow, due to the lack of immediately required additional in-formation or significant masking of the searched object.

The purpose of this experimental study is to develop a methodology for organizing experiments on the selection of applicants for operator positions in information search and similar systems for processing images of scenes of real work situations.

The paper contribution is given as follows:

- The model of the human-machine interface as implementation of reaction to the detected object is proposed. This model is presented as dynamic systems, that al-lows the simulation of the next state of the operator personnel.
- A novel method for the analysis the influence of the flow of micro stressors on operator activity is developed. The sequence of images at discrete moments of time is generated. The operator is exposed on the monitor screen to a sequence of test images with objects of attention of a given class and which operator must implement the corresponding solution. The moments of their exposure and the decisions made by the operator are recorded, and their values are included in the research protocol. Next, hierarchical clustering is used for stress resistance clustering.

The effect on the reaction of a human operator to a deficit or an excess of provided information when making responsible decisions is studied in McEwen and Akil (2020). The model of the operator's stay and exit from stress is considered in Sahin et al. (2019), and the occurrence of neuropsychological overstrain is also explained here. The theoretical foundations of the psycho-diagnostic of stress and methods related to the psychological diagnostic procedure, ethics and stages of the psychological and diagnostic examination are given in Barak and Tsodyks (2023). Various aspects of the influence on the formation of the reliability of operator activity, including the working environment, the functional state of the operator and the intensity of work, are given in Brown and Anderson (2019).

The article Paul and Dykstra (2017) discusses the features of computer training of operators of continuous technological processes in comparison with other subject areas. The possibility of automating the measurement of the properties of the operator's attention using Schulte's tables is confirmed in Kryvenchuk et al. (2019). In Lee (2016) statistical methods of testing results analysis are considered, and the simplest and necessary procedures for statistical processing of knowledge testing results and test quality assessment methods are also presented. The research conducted in Bookstaber et al. (2014) summarized the structure of the stress test and analyzed the methods of generating shock scenarios.

The purpose of the article Battiston and Martinez-Jaramillo (2018) is to study the organizational aspects of stress testing and to determine the place and functions of the supervisory body in the process of stress testing. The methods of quantitative and integral assessment of personnel stress in the process of knowledge verification during attestation are analyzed in Iannello et al. (2017) and DeMenno (2022).

In Allen and Kessel (2002), three strategies for the development of detection systems are proposed, which are based on the principle of balance for purposeful improvement and design of detection systems. As shown in Chapelle (2009), the automation of the process of knowledge control, and the development of computer testing systems is an urgent task.

In work Chou (2000), an analysis of new information and computer training technologies for the development and use of existing interactive educational and training complexes of small arms was carried out. The article McGlohen and Chang (2008) discusses the problems of implementing testing using information and communication technologies. A systematic analysis of organizational problems of the development and application of computer testing technologies in higher education to control students' knowledge was carried out.

The article Zhu et al. (2019) presents an example of realistic image recognition using both manual and automated testing with a decision table. The article Ramanathan et al. (2016) discusses the principles of personnel selection and the most common traditional and non-traditional methods. The material for familiarization and assimilation of the personnel evaluation system to confirm his competence and responsibility is given in Raskin and Kircher (2014). The main theoretical principles and practical aspects of the application of psychophysiological testing to determine the level of professional suitability of polygraph operators are substantiated.

The conceptual contribution of the most valuable reviewed articles is given in Table 1.

**Table 1:** References review.

**References**	**The main contribution**	**Value**
Paul and Dykstra (2017)	Cyber operations stress approaches comparison	The work describes the impact of stress on a cyber security worker. Studying the impact of frustration, mistakes on the work of an employee who uses a computer simulator or just a computer helps to understand various states, causes and consequences.
Zhu et al. (2019)	A method for testing intelligent Applications.	Testing smart applications provides insight into how to manually and automatically recognize certain elements. Which approaches are used by machine or human and how can automatic and manual approaches be combined.
Barak and Tsodyks (2023)	Mathematical models of learning in neurobiology	The article describes mathematical models and how they are used in neurobiology. Also, methods of applying data analysis to study the behavior of simulator operators.
Sahin et al. (2019)	Mathematical model of human behavior for simulating evacuation of buildings during emergencies	The author talks about modeling the behavior of people in evacuation situations. Evacuation gives stress to a person. Therefore, the simulation of such situations is related to the simulation of the microstress situation during the operator's work.

The literature review analysis shows the lack of experimental studies related to person's stressful conditions during working time. In general, the research was carried out after the recipient had completed the work. By comparing the work process and psychophysiological indicators, researchers conclude the existence of a person's stressful state and analyze the results of the impact of existing irritants. For gaps filling, the model of human-machine interface will be developed in the paper.

## 2. Materials and methods

### 2.1. The model of the human-machine interface

One of the identified large numbers of various tasks of operator activity in computerized workplaces is the task of searching for objects of a given class on the images provided on the monitor and making appropriate decisions when receiving them. With this person and the computer function as a single system in the existing working environment. From the point of view of the general mathematical theory of the system, its descriptive model can be presented as follows. Let ***P***(***t***) is some technological process that is managed by a human operator over time [0, *T*_0_ ] where ***T***_**0**_**=**{***t***_***i***_**:*t***_**1<**_***t***_**2**_**…*t***_***N***_ ***i***∈***T***}, and *T*- real time. The essence of its management is reduced to the functions of solving specific problems by the operator, for example, by identifying some object on the presented image of the scene and making the appropriate decision. An example of such processes is the search for objects of a given class on the image of the controlled territory, in the sequence of images of scenes, elimination of deviations in the parameters of the controlled process, search for the necessary data in document databases, editing of texts, etc., presented by their appearance on the monitor.

Change information about the process ***P***(***t***) happens on the monitor screen in moments ***t***_***i***_ **∈*T***_**0**_. The operator by focusing attention and visual analysis of the given set of images ***X***={***x***_***i***_**:*x***_**1<**_***x***_**2**_**…*x***_***N***_ ***i***∈***N***}, using his skills, experience and knowledge tries to identify the desired object in these images. As a result of the search and detection of such an object, the operator then chooses, considering the possible consequences of the decision made, makes an appropriate decision from a set of decisions ***Y***={***y***_***j***_**:*y***_**1<**_***y***_**2**_**…*y***_***N***_ ***j***∈***M***}. Images of situations on the monitor contain all information about the state of what is observed with the help of the monitor and obviously the corresponding technical means of the controlled process ***P***(***t***).

In the process of its activity, the operator can be in different functional states ***C***=***C***(*c*_***i***_**)**, in psychophysiological states from normal to nervous overstrain. These states can be caused both by the influence of the external environment and by the provided information. In other words, in the absence of influence from the work environment, the functional state of the operator is a function of the provided image of the situation that occurred in the process ***P***(***t***), namely the function ***C***=***C***(*c*_***i***_**)**. It is this function that reflects the personality of the operator in the model and was used to study the activity of the operator in a state of micro stress.

The basis of this process is the concept of a system, that is a chain: “process → monitor → operator → computer → decision”, which makes up the system together. In set-theoretic terms at this level, the system can be simple and quite naturally defined as a product ***S***⊂***X***×***Y***.

In the sense of model development plan the sets ***X*** and ***Y*** differ from each other in their position on the time axis, they are subject to the moments of time of their implementation. Elements ***x*** **∈*X*** depends on time $X=\left\{x:x_{i}\equiv x\left(t_{i}\right),\;{\;t}_{i}\in T,\;i=\bar{1,N}\right\}$, but decision elements $Y=\left\{y:y_{j}\equiv y\left(t_{j}\right),\;{\;t}_{j}\in T,\;j=\bar{1,M}\right\}$ always delay by ***t***_***i***_, which corresponds to the total duration of search, detection, analysis, selection and decision-making until the moment of its implementation by the relevant team, i.e., ***t***_***j***_**=*t***_***i***_**+Δ*t***_***i***_.

The average time of this delay can be used as an estimate of the efficiency of a particular operator during his shift. Over time, the efficiency and accuracy of the operator deteriorates. Each operator has its own optimal change and maximum possible change.

Since the human-machine interface is a dynamic system (Sahin et al., 2019), it makes sense to consider the behavior of the operator depending on the input information. In this general plan, we are talking about his reaction to the detected object, more precisely, the efficiency of detecting the object and making a decision. To establish the relationship between the input and output of the system, which refers to different moments of time, the general mathematical theory of systems introduces the concept of the system's response to an input stimulus (Paul and Dykstra, 2017). In other words, for each operating situation, for each image on the screen for an operator in a normal functional state, there are two such displays:

- making a decision

$$\bar{\rho }=\left\{\rho_{t}:C_{t}\;\times \;X_{t}\;\to \;Y_{t}\;\&\;t\;\in \;T\right\}$$

- transition to another state

$$\bar{\varphi }\;=\;\left\{\varphi_{tt^{\prime }}:\;C_{t}\;\times \;X_{tt^{\prime }}\;\to \;C_{t^{\prime }}\;\&\;t,\;t^{\prime }\;\in \;T\;\&\;t^{\prime }\;>\;t\right\}.$$

These reflections can be explained as follows. Being at the moment of time ***t***∈***T*** in the state of ***C***_***t***_, the operator perceives the image of the ***x***_***t***_**∈*X*** and detects the desired object on it, analyzes the situation, and makes a decision of ***y***_***t***_**∈*Y***, spending the necessary time normally, without changing his functional state (there is no neuropsychic tension).

If the provided image turns out to be such that it is not possible to immediately find the desired object, and the time limit for the search is limited, the operator can switch from a normal state to a nervous state, continuing the search for the object. If we assume that at the moment *t*∈*T* the operator has not detected the object and feels that the exposure time is running out, he is aware of the complexity of the situation, and at this moment he can get a shock. This shock can be interpreted as stress or micro stress. The double time index means the time interval ***t***^′^**>*t*** during which the operator passed from a normal to a neuropsychical state.

In general, the model of the human-machine interface as such an information and search system can be represented by a tuple ***S***=〈***X***,***Y***,***C***,*ρ*,*φ*,***T*** 〉.

The use of the mathematical apparatus of set theory provides an opportunity to optimize the organization of research, since sets are presented quantitatively, the mappings of which are considered functionally and all of them are considered in time. In addition, based on such a model, it is possible to create a sufficient number of images that simulate the scenes of almost any real operator activity.

### 2.2. Method for the analysis the influence of the flow of micro stressors on operator activity

The localization of the object of attention on the test images is random, and the ratio of its size to the size of the test image practically amounts to several orders of magnitude (by area). In turn, individual fragments of the image, namely the locations of objects, can be both homogeneous, one-colored, smooth, and contain various other objects. These other objects are related to the background and can be larger or smaller than the object of attention and have an irregular shape. They significantly impair the visibility of the object of attention and are actually obstacles to the search. In the presence of such obstacles, the search process becomes more complicated, requires increased concentration of attention, creates for the operator a certain visual discomfort at first, which turns into additional neuropsychological stress or stress.

The sequence of such images at discrete moments of time reflects the situation observed by the operator, who should make the appropriate decision in case of detection an attention object. On a homogeneous background, the attention of object detection is reduced to a simple reaction of object recognition, however, in the presence of obstacles, noise, the time of such a reaction–searching for an object, will increase depending on their intensity. This, in turn, gives reason to say about the images that some of them have greater and others less difficulty in detecting the object.

On the other hand, individual characteristics, such as groups of operators, will show that the detection time of the object of attention on the same image will be different. Obviously, the more complex the image, the longer the average time to detect an object in this image. So, in this case, the category “complexity” is a subjective characteristic, but in general it has a certain dimension, namely the duration of the search time from the moment of providing the image to the moment of its discovery. The value of the indicator of the complexity of a particular image is the average value of the duration of the search, determined by the data obtained by different operators and in different experiments. The only requirement for this determination of the value of this indicator is the homogeneity of the group of “expert recipients” in terms of their training levels, skills, etc. In other words, the complexity of such images is determined by the time of searching and detecting the object of attention of a given class. The objectivity of such an assessment will be higher the more homogeneous (in terms of training) the group of “expert recipients” is.

### 2.3. Selection of test images

Let a group of operators be the subject of research, and the purpose of the research is to determine individual stress resistance. The preparation and conducting of the experiment were carried out as follows.

#### 2.3.1. Creating a basic sequence of test images

At this stage, they create test images and form them into a sequence. The images in this sequence simulate the background of the working scene, on which the researcher manually or with the help of random numbers determines the coordinates of the places where the objects will be located. The placement of the object on some images should be quite easy to detect, and on others it should be difficult, i.e., hidden, but so that it can be found. An important point here is that all the details of the object were not closed, the object should look whole. After placing the objects, the received test images now have this status, they are formed into a sequence of scenes, mostly randomly. This sequence is shown to each of the group of recipients, fixing the time of processing each of the images by each recipient. At this stage, the duration of the experiment is determined, the number of test images in a sequence, from which the exposure time of the images is determined.

**Figure 1:**
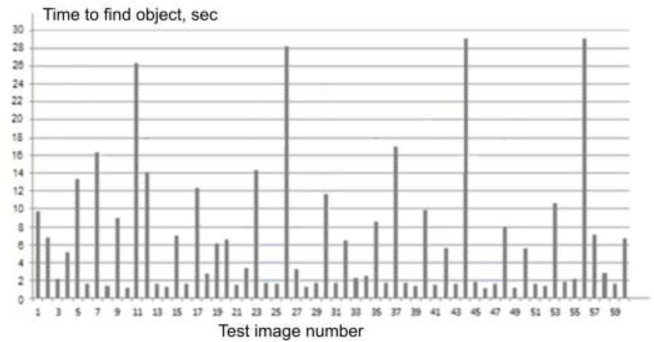
Search time for objects in the basic sequence.

The duration of such an experiment can be determined depending on the contingent of recipients, for example, for professional operators, it is the duration of a shift or its part (the second half), and for young people, it is the duration of one or two academic hours. Depending on the complexity of the images of the scenes, exposure of the test image is up to 1–2 min. In the experiments conducted by the authors, the sequence included 180 test images with exposure of each of them for 30 s (Figure 1).

#### 2.3.2. Determination of search complexity of test images

The sequence formed in this way is included in the process of professional selection or training of a group of recipients. The task is set and explained to them in advance, and if possible, an imitation of a real workplace and environment is carried out. After the “start” command, test images appear on the monitor screen, the operator, having detected the specified object, presses the corresponding key. When the test image appears, the stopwatch starts, and when the key is pressed, the operator stops it. As a result, the computer records the processing time of this image by the operator and resets the stopwatch.

All applicants work with the same sequence. As a result, for each test image, a sample of time values spent on them by each operator (recipient) will be obtained. The average value of the time spent by them on processing a particular test is actually a time indicator of their search complexity. Since these time indicators are quantitative values, for them it is possible to enter the corresponding scale of search complexity for test images of only this sequence and this group of recipients. Thus, each image will be characterized by its indicator. Figure 1 shows a diagram of times for searching for an object of attention on some subset of test images.

#### 2.3.3. The sequence of stressful events

**Figure 2:**
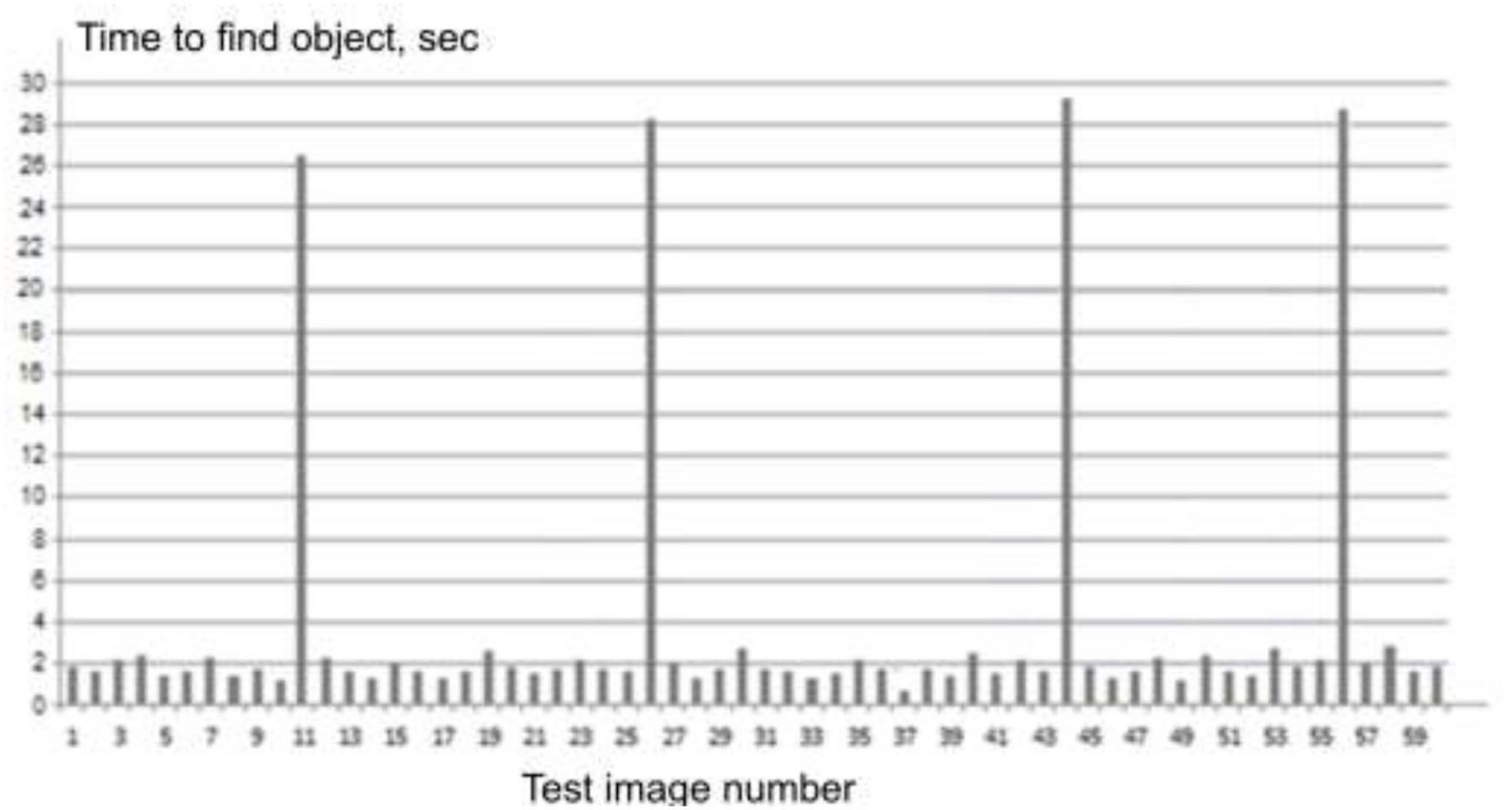
View of the experimental sequence.

If we assume that the operator has been processing images of rather low complexity for some time, object detection is easy, and he has adapted (used to) this mode of presenting test images. If an image of high complexity suddenly appears and it was not possible to immediately detect the object, he will be forced to activate and concentrate his attention on the search, which in the mode of time shortage, for example, limiting the exposure to 20 s, will cause him (although maybe not) a micro-stress situation. In this regard, it is of interest to form such a sequence, which will consist of test images of low complexity and several images of high complexity are included in it (Figure 2).

Therefore, after the search complexity of the base sequence images is determined, the images with the lowest search complexity are selected, for example, the test images with the smallest search time of <2 s and the longest search time of >18 s. Next, a new basic sequence is made from the images that have minimal complexity (they can be repeated, but not one after the other). This new sequence includes, following the principle of rare events, test images whose search complexity exceeds 18 s. Obviously, all the images in this sequence must be randomly arranged. The view of a fragment of such a sequence is shown in Figure 2. The value of the duration of such micro stress can be represented by the appropriate mathematical model of general stress.

### 2.4. Options for presenting test images

In general, the organization of experimental research can be presented as follows. Let the human operator be exposed on the monitor screen to a sequence of test images with objects of attention of a given class and which the operator must identify and implement the corresponding solution. The moments of time of their exposure and the decisions made by the operator are fixed, and their values are included in the research protocol.

**Figure 3:**
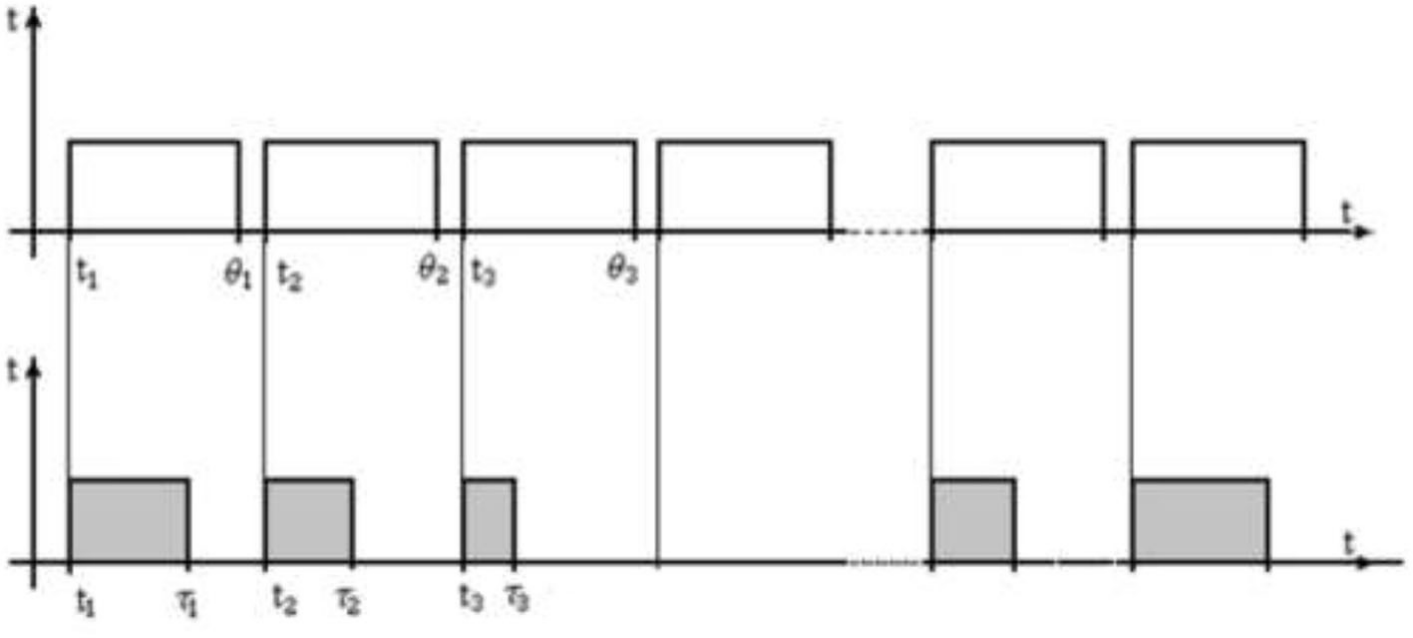
Regular stream of test images. Where *t*_1_, *t*_2_, … is starting point, and θ_1_, θ_2_, … are moments of exposure termination; τ_1_, τ_2_, … are moments of decision-making by the operator.

The main point here is the method of exposure of the test images. So, for the organization of experimental studies, the following three options can be specified for the method of providing test images to the operator on the monitor. Each of them includes two streams of rectangular pulses, synchronized in time along the pulse front. In the first version in Figure 3, the upper sequence corresponds to a regular stream of test images, marked by light rectangular pulses of the same duration and amplitude. Here the exposure of the tests is carried out at the same time intervals. The images of the situations are presented in a regular sequence (Figure 3).

The flow of test images in Figure 3 is a part of the experimental study. The lower sequence of dark impulses corresponds to the duration of the search for the object of attention, its detection as the object being searched for, and decision making. In other words, this sequence reflects the results of the human operator. Each slice of the pulse of this flow corresponds to a reaction-result as the moment of decision-making by the operator. If the operator did not find the desired object in the image, then the dark pulse will be absent in the bottom sequence. In such a graphic presentation, the upper and lower pulses are synchronized along the edge, at the moment of the appearance of the test image, the stopwatch is turned on, which is automatically turned off at the moment of the operator's decision.

**Figure 4:**
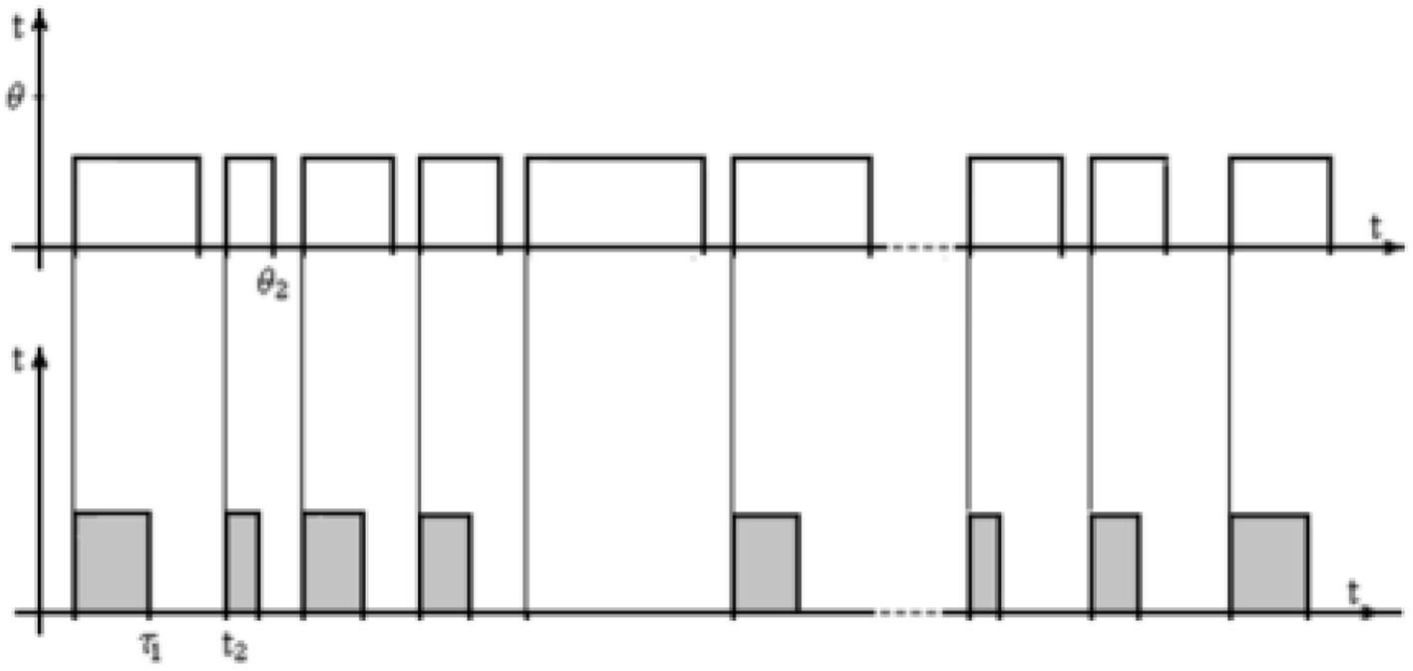
Irregular flow of test images.

In the second version, shown in Figure 4, the exposure of the test images is irregular and has different durations, but the pulse fronts of the upper and lower sequences are also synchronized. In the general case, when using an irregular flow, the duration of the pause between the upper pulses can be different. In addition, a variant is possible when the duration of the pulses of the upper and lower sequences are the same, and the duration of the pauses can be different. This means that after the operator makes a decision, the test image disappears, and the next one appears at the next moment.

**Figure 5:**
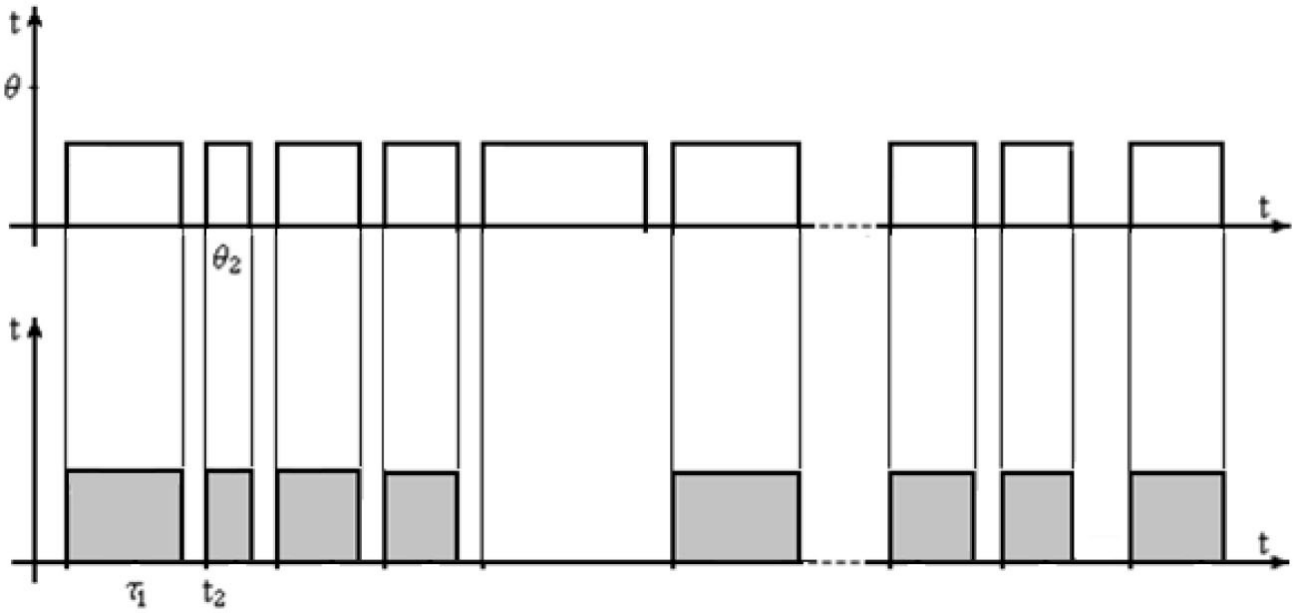
Irregular flow of decision-driven test images.

In the third option, shown in Figure 5, the exposure of the image continues until the decision is made, after which the next image is displayed on the screen. The last option greatly reduces the duration of research with a limited image base since the duration of the exposure is equal to the duration of the search. In a certain sense, this option has a positive value, especially for laboratory research since it allows you to process more test images during the same experimental period.

The considered options for providing test images and fixing decisions are not equivalent from a psychological point of view. The fact is that with regular exposure, when the operator has very quickly identified the object and made a decision, he has some time left before the exposure of this image is completed. This causes a certain relaxation, and at the moment of the appearance of a new image, the operator sharply mobilizes attention and goes into a tense state. Such an irregular change in concentration and relaxation is a negative element in operator activity. On the other hand, when the next image is presented immediately after making a decision, constant nervous tension is created for the operator due to the need to maintain concentration all the time, which is also negative. Therefore, the choice of one or another mode of providing test images on the monitor screen is not trivial, especially for long-term experiments. Display of experimental research with the help of impulse flows provides a mathematical formulation of experiments and presentation of results by mathematical models of time series, in particular exposure of test images and object detection and decision-making.

## 3. Results

Nine recipients which are student operators took part in the experimental research. The experiment was conducted in two stages: in the first stage, the search complexity of the given set of test images was determined, and in the second stage, a new sequence was built. The initial volume of the sequence was 180 images of the tests, which were exposed on the monitor screen with a maximum duration of 30 s according to this rule. If the operator detected a given object on the test image in <30 s, the given test image was immediately replaced by the next one. In the event that the object was not detected on a given test image within 30 s, then it was replaced by the next one from this sequence.

In the sense of stress resistance, an assumption is made–if the detection time exceeds 2/3 of the exposure time or if the operator did not detect the object during the exposure time of the test image, then it can be assumed that it was in a state of micro stress. Although such categorization may be wrong, in this case, the authors consider it permissible.

The value of the time from the moment of the appearance of the test image to the moment of decision-making was used as an assessment of the search activity of the operator. In addition, to understand the stress state, the exposure time of test images is divided into two parts: the first 1–20 s and the second 20–30 s. The search time was recorded in milliseconds. The procedure for providing images corresponded to the third variant of the impulse flow model, that is, as soon as the operator made a decision about the detected object, the next one from the given sequence was immediately exposed. The volume of the provided sequence was 180 test images. The individual results of the experiments are given in the Table 2. This table presents only correct response.

**Table 2:** The individual results of the experiments.

**Parameter**	**Operator s**								
	**1**	**2**	**3**	**4**	**5**	**6**	**7**	**8**	**9**
*a*	10	16	11	18	10	15	11	8	15
*b*	17	10	14	13	11	9	7	13	77
*x*	24.8	24.1	23.2	23.9	24.2	24.5	24.0	24.4	24.8
*Min*	20.9	20.3	20.2	20.5	20.2	20.2	20.6	20.9	20.1
*q*1	23.2	21.6	20.6	22.4	23.2	21.9	21.6	21.8	23.8
*Mode*	24.5	24.3	22.6	23.0	24.2	25.2	24.7	23.5	25.0
*q*3	26.2	26.1	24.7	25.6	25.9	26.4	26.0	27.8	26.5
*Max*	29.1	29.3	29.6	29.8	27.7	29.4	26.9	28.4	28.3

The following parameters are used: *a*–the number of detected objects within the stress time; *b*–the number of missed objects; *x*–average stress time. Parameters: min, *q*1, *mode*, *q*3, max are parameters of the stress time interval boxplot diagram, namely extremes, quartiles, mode.

**Figure 6:**
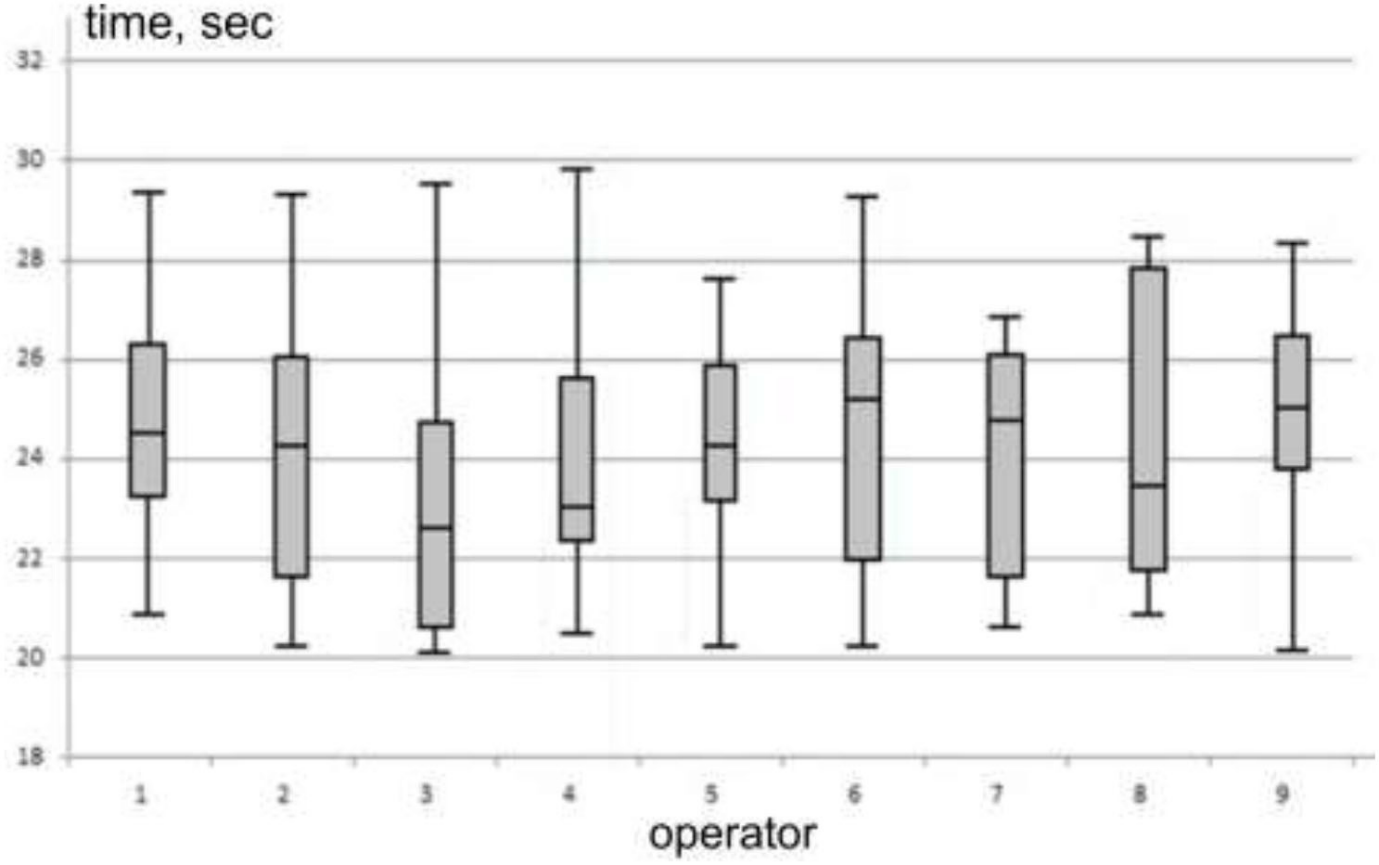
Image of individual boxplot diagrams.

Using the parameters of the descriptive statistics of the results of the experiments for each of the recipients in Figure 6 shows constructed boxplot diagrams for the studied group of recipients, as a result of which the general structure of the distribution of individual data is determined.

As the resulting charts show, with the exception of recipient 3 and almost 9, within the interquartile range are symmetric, with recipients 1, 4, 5, and 8, and recipients 2, 6, and 7 having left- and right-sided asymmetries, respectively. This means that more of the rectangle has more variance. It can be assumed that intuitively feeling the completion of the exposure of the test image increases excitement and a certain, mostly significant, nervous tension. The authors assume the following regarding the asymmetry of distributions. For a left-sided distribution, the upper part of the rectangle is larger and corresponds to values greater than the mode value. Their spread between the mode and the third quartile is larger, and therefore it can be assumed that these recipients are characterized by a delayed reaction. For right-sided asymmetry, on the contrary, the lower part of the rectangle corresponds to the values of the search time, which are smaller than the values of the mode, they are characterized by haste of the reaction.

The obtained boxplot diagrams are individual statistical displays of the characteristics of the operators within the stress time interval. These individual characteristics, according to the given table, show the quality of search activity of operators, namely the number of missed and detected objects in the stressful time zone.

When constructing the boxplot diagram, the calculation of emissions was not carried out, since the interval of values here is limited by the condition of 20–30 s. From Figure 6, it can be pointed out that if we place individual boxplots relative to fashion in ascending order, we will get their rating (Figure 6).

During the laboratory study of the formed group of operators, the task of dividing them into subgroups based on close individual indicators arises. Given the small number of classification features and the number of recipients, hierarchical agglomerative cluster analysis was used to determine subgroups. The following individual values of the recipients were taken as signs: the number of detected objects within the stressful time; the number of missed objects; mean stress time, as well as the minimum and maximum time value in the stress time interval, the first and third quartile values, and the mode value. Therefore, the parameters of the boxplot diagram of the stress time interval are taken as the values of the classification features.

## 4. Discussion

**Figure 7:**
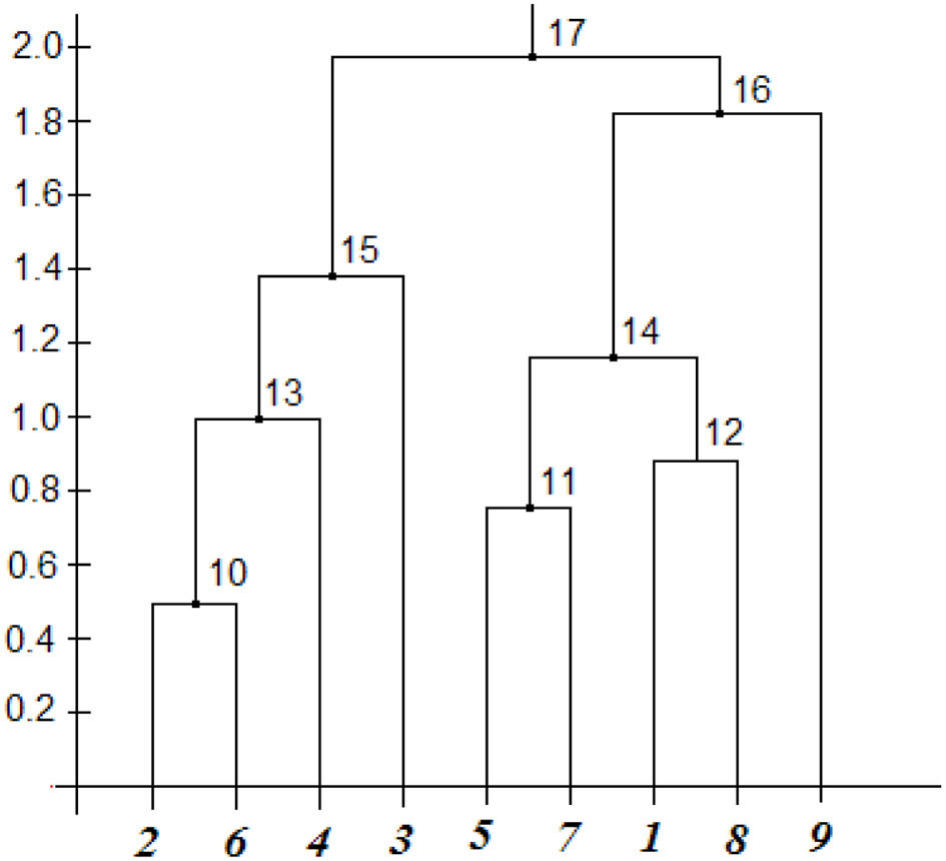
Results of the cluster analysis of the group of recipient-operators.

For the clustering procedure, the values of the classification features were reduced to the interval [0, 1] and a flexible strategy was chosen, and the Euclidean metric was used to construct the proximity matrix. The features are normalized for each parameter. The result of the cluster analysis, in the form of dendrogram parameters, is shown in the Table 3 in Figure 7 on the left, which shows the results of combining objects and the distances by which the combination was performed, and the cluster analysis dendrogram itself is shown in this figure on the right.

**Table 3:** Results of the cluster analysis of the group of recipient-operators.

**Combining objects**	**Association number**	**Distance between objects**
**Dendrogram parameters**		
2 + 6	10	0.494
5 + 7	11	0.746
1 + 8	12	0.883
10 + 4	13	1.000
12 + 11	14	1.158
13 + 3	15	1.384
14 + 9	16	1.822
5 + 16	17	1.974

Visual analysis of the dendrogram gives the following result.

1. At level 1.5, you can visually distinguish three clusters that include such recipients:

- cluster 1: it includes recipients 2, 6, 4, 3;
- cluster 2: it includes recipients 5, 7, 1, 8;
- cluster 3: it only includes recipient 9.

Moreover, the first and second clusters are similar to each other in terms of the homogeneity of recipients, as evidenced by the distance of associations between objects in them, which lies within 0.6–1.4.

2. Recipients vary greatly in their individual characteristics.

For example, the smallest distance between operators 2 and 6, which is equal to 0.5 on the scale of the dendrogram and is 25% percent of the total scale. Pairs 5 and 7 and 1 and 8 differ even more from each other and from pair 2 and 6. Recipients 4 and 3 are very different from pair 2 and 6 even though they all belong to the same cluster.

3. Cluster 3 is located separately and as far as possible from the first two, which is quite clear from the analysis of its performance indicators.
4. The distance between these clusters is almost the same and is in the range of 1.822 - 1.974, it can be argued that the subgroups are significantly different from each other.
5. If the division into clusters is carried out at the level of 0.9 - 0.95, it is possible to distinguish three clusters of two operators and three clusters of one operator, although even in these pairs the operators differ quite noticeably.

In this way, the division of the group of operators into professionally homogeneous groups using a computer simulator, the development of image-tests, scenarios provide a sufficiently objective assessment of the results of professional selection, conducting relevant training. In addition, personnel attestation can be carried out in the same way using control materials.

The mentioned before method allows us to find the individual parameters of the recipients such as:

- the recipients in stressful situation;
- mean stress time;
- the minimum and maximum time value in the stress time interval.

Therefore, these parameters are further used to classify recipients according to the level of stress resistance. The developed method can be used for detecting the attention level.

## 5. Conclusions

Conducting experimental research, especially for the purpose of studying human properties, is a very complex procedure that requires significant preparation and organization, including task development and problem setting. This requires in-depth knowledge of this real operator activity, minimizing the difference between the real operator activity and its reproduction on the simulator. In addition, an important point is the completeness of the received information (data) and their detailed interpretation.

The obtained experimental data gave grounds to objectively assess the qualifications of the recipients, both in the individual plan–the parameters of the boxplot diagram, and in the professional–the parameters of the dendrogram of the hierarchical agglomerative analysis. The latest results of the division of the group of recipients showed the closeness of their characteristics and the possibility of their professional selection for operator specialties.

The given mathematical model of operator activity in information search systems formally describes the operator's work, in particular by the function of transition to another functional state, since the operator is with the same image that led to his stress. The conducted experimental study with nine recipient-operators, according to the scenario in the form of a sequence of test images provided on the monitor screen, provided the result in the form of reactions to micro-stresses. With the help of mathematical processing methods: descriptive statistics, determination of quartiles, construction of boxplot diagrams and hierarchical agglomerative cluster analysis, an objective grouping of this group of recipients was obtained. In this study, each operator is represented by an individual boxplot diagram, and the group of operators is divided into subgroups according to individual test indicators. This made it possible to carry out an objective professional selection for the formation of camera personnel.

The limitation of current work is the ability to deal with micro-stresses but not with panic attacks. This limitation is related to the used dataset and should be investigated separately. In addition, after-effect of micro-stressor should be investigated separately.

## Data availability statement

The raw data supporting the conclusions of this article will be made available by the authors, without undue reservation.

## Author contributions

Conceptualization, resources, writing—original draft preparation, and supervision: RK. Methodology, formal analysis, writing—review and editing, project administration, and funding acquisition: NS. Software, validation, investigation, data curation, and visualization: BK. All authors have read and agreed to the published version of the manuscript.
